# Assessment of potential pathogenic bacterial load and multidrug resistance in locally manufactured cosmetics commonly used in Dhaka metropolis

**DOI:** 10.1038/s41598-023-34782-9

**Published:** 2023-05-13

**Authors:** Namira Nusrat, Maftuha Ahmad Zahra, Akash Ahmed, Fahim Haque

**Affiliations:** Microbiology Program, Department of Mathematics and Natural Sciences, BRAC University, Dhaka, Bangladesh

**Keywords:** Antimicrobial resistance, Pathogens, Microbiology, Bacteriology

## Abstract

In Bangladesh cosmetics are being produced disregarding the Good Manufacturing Practices. So, this study aimed to test the level and nature of bacterial contamination of such cosmetics. A total of 27 cosmetics comprising eight lipsticks, nine powders, and ten creams were bought from New Market and Tejgaon areas of Dhaka city and tested. Bacteria was detected in 85.2% of samples. Majority of the samples (77.8%) exceeded the limit given by the Bangladesh Standards and Testing Institution (BSTI), Food and Drug Administration (FDA) and the International Organization for Standardization (ISO). Both Gram-negative (*Escherichia coli*, *Pseudomonas aeruginosa*, *Klebsiella pneumoniae* and *Salmonella spp.*) and Gram-positive bacteria (species of *Streptococcus*, *Staphylococcus*, *Bacillus* and *Listeria monocytogenes*) were identified*.* Hemolysis was observed in 66.7% Gram-positive and 25% Gram-negative bacteria. Multidrug resistance was tested in 165 randomly selected isolates. Every species of Gram-positive and Gram-negative bacteria exhibited varying levels of multidrug resistance. The highest levels of antibiotic resistance were in broad-spectrum antibiotics (ampicillin, azithromycin, cefepime, ciprofloxacin and meropenem) and narrow-spectrum Gram-negative antibiotics (aztreonam and colistin). Multidrug resistance was 12–78% in Gram-negative bacteria and 12–100% in Gram-positive bacteria. Coagulase and DNase were identified in 97.5% and 5.1% of *Staphylococcus aureus* isolates respectively. Our findings indicate that these cosmetics pose a risk to the public’s health.

## Introduction

Cosmetics are used by everyone worldwide to enhance their hygiene as well as their beauty. Complete sterility is not mandated during the use of cosmetic products or even in unopened cosmetic products, but microbially contaminated cosmetic products can cause various infections^1^. As microbial contamination is capable of causing health problems, it is vital to guarantee that cosmetic products as well as their raw materials are manufactured according to the guidelines of Good Manufacturing Practices, Bangladesh Standards and Testing Institution (BSTI), the International Organization for Standardization (ISO) and the Food and Drug Administration (FDA) so they do not cause harm to the skin of consumers^2^. For non-eye cosmetics, the level of contamination of cosmetics should not exceed 10^3^ CFU/g or ml; for eye area cosmetics, mucous membranes, and children < 3-year-old, the level of contamination should not exceed 10^2^ CFU/g or ml. These standards are according to the FDA and International Organization for Standardization ISO 17516:2014^3–5^. Both the European Union and Bangladesh are members of International Organization for Standardization (ISO). Also, according to ISO 17516:2014 guidelines *Escherichia coli*, *Pseudomonas aeruginosa*, *Staphylococcus aureus,* and *Candida albicans,* i.e., organisms that potentially pathogenic must be completely absent in 1 ml or 1gm of the product^4,5^. According to the Bangladesh Standards and Testing Institution (BSTI) the cosmetic manufactured does not need to be sterile at the end but the bacterial count should not exceed 1000 microorganisms/g. No pathogenic bacteria should be detected in cosmetics at any level^6^.

Reports of microbial contamination of commercially available products have been reported in scientific literature. *Pseudomonas fulva, Pseudomonas monteilii, Citrobacter freundii, Staphylococcus aureus, Staphylococcus spp.* and *Candida spp.* have been isolated in lipsticks^2,7–9^. In powders, bacteria such as *Bacillus spp., Staphylococcus aureus, Staphylococcus epidermidis, Salmonella,* and *Pseudomonas spp.* have been identified^2,10,11^. In creams, *Escherichia coli, Bacillus spp, Bacillus cereus, Klebsiella spp., Klebsiella pneumoniae, Pseudomonas spp., Staphylococcus spp., Enterobacter spp., Enterococcus faecalis, Micrococcus spp., Staphylococcus aureus, Streptococcus pyogenes,* and *Enterobacter aerogenes* have been detected^2,8,10,12–15^. Another study has shown that *Escherichia hermannii, S. aureus, Bacillus cereus,* and *Enterobacter* species were isolated from lip glosses and lipsticks. This study also showed the presence of *Buttiauxella agrestis,* which had never been isolated before in cosmetic products. It was found in a sample of hair relaxer^16^.

Bacterial contamination of products can cause human illness. Some are mild, like conjunctivitis and allergy; others are more severe, like systemic keratitis, blood infection and whole-body inflammation^17^. Even in some cases, cosmetics infected with bacteria have caused death^18^. According to several studies, *Staphylococcus* was the most common bacterial skin pathogen^19–21^. A study has also determined a connection between conjunctivitis, impetigo, and *Staphylococcus aureus*^22^. According to a survey conducted^23^ several women had symptoms of bacterial blepharitis and were infected with large concentrations of *Staphylococcus epidermidis.* This was isolated from both their eye cosmetics and the corner of their eyes.

The presence of drug-resistant bacteria has been reported in various studies. *Pseudomonas aeruginosa, Escherichia coli, Staphylococcus aureus, Chromobacterium violaecium* and *Listeria monocytogenes* were observed to be resistant to both broad-spectrum and narrow-spectrum antibiotics^24,25^. Drug-resistant pathogens have also been detected in baby products such as baby lotion, where *Enterobacter gergoviae, Serratia marcescens, Pseudomonas aeruginosa*, and *Enterobacter cloacae* bacteria were isolated and found to be resistant to both broad-spectrum and narrow-spectrum antibiotics^16^.

In Bangladesh, local cosmetics are manufactured, where Good Manufacturing Practices are not maintained. These cosmetic factories are in Chawkbazar area of old town Dhaka. These cosmetics are then distributed to various areas such as New Market. It is the main distribution point, and it supplies cosmetics to retail shops all over the city. The study focused on buying products from the main distribution point as opposed to going to different locations to purchase the same cosmetics^26,27^.

Bangladesh reached lower-middle income status in 2015. It is on track to graduate from the UN’s Least Developed Countries (LDC) list in 2026^26^. This study informs how quality is ensured in luxury products in low- and middle-income countries. The cosmetics tested in the study were all purchased at a bargain price. Also, some of the products were dupe products pretending to be original. This practice of manufacturing low-quality products with the packaging of famous international brands is quite common in Dhaka^27,28^. These local cosmetics are being sold in the capital and other places under the guise of popular foreign and local brands, so many people are misled and purchase them^27–29^. Customers belonging to lower income households purchase these products as they are comparatively cheaper. There is a high chance that these cosmetics might contain pathogenic bacteria that can cause serious infections. People have reported various health problems, such as eye infections, allergic reactions, skin rashes, swollen lips, and chemical burns from using these products^30^. According to Dr. AK Lutful Kabir, associate professor of the Department of Pharmaceutical Technology of Dhaka University, adulterated cosmetics could also reach the blood through the skin and could even cause cancer^27^. As mentioned previously Bangladesh is a least developed country and such diseases pose an economic burden to the patients. In a study conducted in 2018 at Birdem General Hospital, Dhaka, 52% of the patients suffering from skin conditions were financially poor^31^. Nevertheless, knowledge of bacterial pathogens isolated from such contaminated cosmetic products is inadequate, as only limited studies have been conducted. For these reasons, the present study attempted to isolate and identify specific bacterial pathogens contaminating the cosmetics and also to determine their antibiotic resistance capability.

## Results

### Total aerobic bacterial plate count of lipsticks, powders, and creams

After processing the samples, 0.1 ml of each sample was spread on Modified Letheen Agar to obtain the aerobic plate count using Eqs. (1), (2) and (3). Among the 27 samples, the total aerobic plate count of 21 samples exceeded the reference value of BSTI, ISO and the FDA. Table 1 shows that 87.5% of lipsticks, 88.9% of powders, and 60% of creams exceeded the reference limit.

**Table 1:** Total aerobic plate count for lipsticks, powders, and creams is mentioned as well as the limit of contamination allowed.

Product type	No. of brands	No. of samples contaminated			Limit of contamination allowed:
		Bacterial load in CFU/g			
		< 10^3^	10^3^–10^5^	> 10^5^	
Lipstick	8	1	3	4	<10^3^ CFU/g
Powder	9	1	2	6	
Cream	10	4	4	2	

Both Gram-positive and Gram-negative bacteria were isolated from the cosmetic samples. *Escherichia coli, Salmonella spp., Klebsiella pneumoniae* and *Pseudomonas aeruginosa* were the Gram-negative bacteria isolated and the Gram-positive bacteria isolated were *Staphylococcus aureus, Staphylococcus epidermidis, Bacillus cereus, Bacillus spp., Streptococcus spp.* and *Listeria monocytogenes*. *Escherichia coli*, *Klebsiella pneumoniae*, *Pseudomonas aeruginosa, Salmonella spp., Staphylococcus aureus, Staphylococcus epidermidis, Bacillus cereus* and *Bacillus spp.* were identified in all three types of products (Table 2). *Listeria monocytogenes* was detected in only powder samples. These bacteria were identified by biochemical tests (Supplementary Table 2). *Escherichia coli*, *Salmonella spp.*, *Klebsiella pneumoniae*, *Staphylococcus aureus*, and *Streptococcus spp.* isolates were further confirmed by PCR (Supplementary Figs. 1–5).

**Table 2:** CFU/ml of different bacteria found in all samples.

Product type	No. of products in which isolates were detected	Bacterial isolates	No. of samples contaminated		
			Bacterial load in CFU/ml		
			< 10^3^	10^3^–10^5^	> 10^5^
Lipstick	6	*Escherichia coli*	1	2	3
	2	*Salmonella spp.*	1	1	0
	2	*Klebsiella pneumoniae*	0	2	0
	4	*Pseudomonas aeruginosa*	2	1	1
	8	*Staphylococcus aureus*	2	4	2
	6	*Staphylococcus epidermidis*	1	3	2
	2	*Bacillus cereus*	0	0	2
	2	*Bacillus spp.*	0	1	1
	4	*Streptococcus spp.*	0	2	2
Powder	6	*Escherichia coli*	1	3	2
	2	*Salmonella spp.*	0	0	2
	4	*Klebsiella pneumoniae*	0	3	1
	3	*Pseudomonas aeruginosa*	0	2	1
	7	*Staphylococcus aureus*	1	2	4
	6	*Staphylococcus epidermidis*	0	4	2
	4	*Bacillus cereus*	0	3	1
	5	*Bacillus spp.*	0	1	4
	5	*Streptococcus spp.*	0	3	2
	4	*Listeria monocytogenes*	2	2	0
Cream	3	*Escherichia coli*	0	1	2
	1	*Salmonella spp.*	0	0	1
	2	*Klebsiella pneumoniae*	0	1	1
	1	*Pseudomonas aeruginosa*	0	1	0
	4	*Staphylococcus aureus*	0	3	1
	1	*Staphylococcus epidermidis*	1	0	0
	2	*Bacillus cereus*	0	1	1
	3	*Bacillus spp.*	0	1	2
	2	*Streptococcus spp.*	1	1	0

The type of cosmetic product as well as the number of cosmetic products that it was found in is mentioned. The name of the bacterial isolates and range of bacterial loads found in the products is also mentioned.

### Hemolysis patterns

The test was performed to check the hemolytic ability of the isolates. The type of hemolysis of each bacterial sample is presented in Supplementary Fig. 6. The percentage of specific hemolysis patterns is presented in Table 3.

**Table 3:** Percentage of observed hemolytic organisms.

Hemolytic pattern	Samples	Percentage of specific hemolytic pattern	Percentage of hemolytic bacteria found in total samples
Alpha hemolysis	Lipsticks	4.76	1.56
	Powders	0	
	Creams	0	
Beta hemolysis	Lipsticks	61.90	61.72
	Powders	56.36	
	Creams	59.1	
Gamma hemolysis	Lipsticks	33.33	36.72
	Powders	43.64	
	Creams	40.90	

The type of hemolysis, percentage of hemolysis seen in different cosmetic products as well as the total percentage of hemolysis is mentioned.

### DNase and coagulase test

As previously mentioned, *Staphylococcus aureus* is a common skin pathogen. For this reason, further screening was done to assess its pathogenic capabilities. In the DNase test, only 5.1% of the isolates were positive. Both isolates were obtained from powder samples. However, for the coagulase test, 97.5% of the isolates tested positive for plasma coagulase-reacting factor (CRP).

### Antibiotic resistance pattern of the isolates

In this study, a total of 165 isolates have been randomly selected to identify the antibiotic resistance of different organisms. All the isolates were subjected to the Kirby-Bauer Disc Diffusion test. The number of isolates tested for antibiotic susceptibility was *Escherichia coli* 25 isolates, *Salmonella spp.* 6 isolates, *Klebsiella pneumoniae* 13 isolates, *Pseudomonas aeruginosa* 10 isolates, *Staphylococcus aureus* 39 isolates, *Staphylococcus epidermidis* 20 isolates, *Bacillus cereus* 13 isolates, *Bacillus spp.* 21 isolates, *Streptococcus spp.* 14 isolates and *Listeria monocytogenes* 4 isolates. All antibiotic susceptibility patterns to specific bacteria are presented in Supplementary Figs. 7–16.

**Figure 1:**
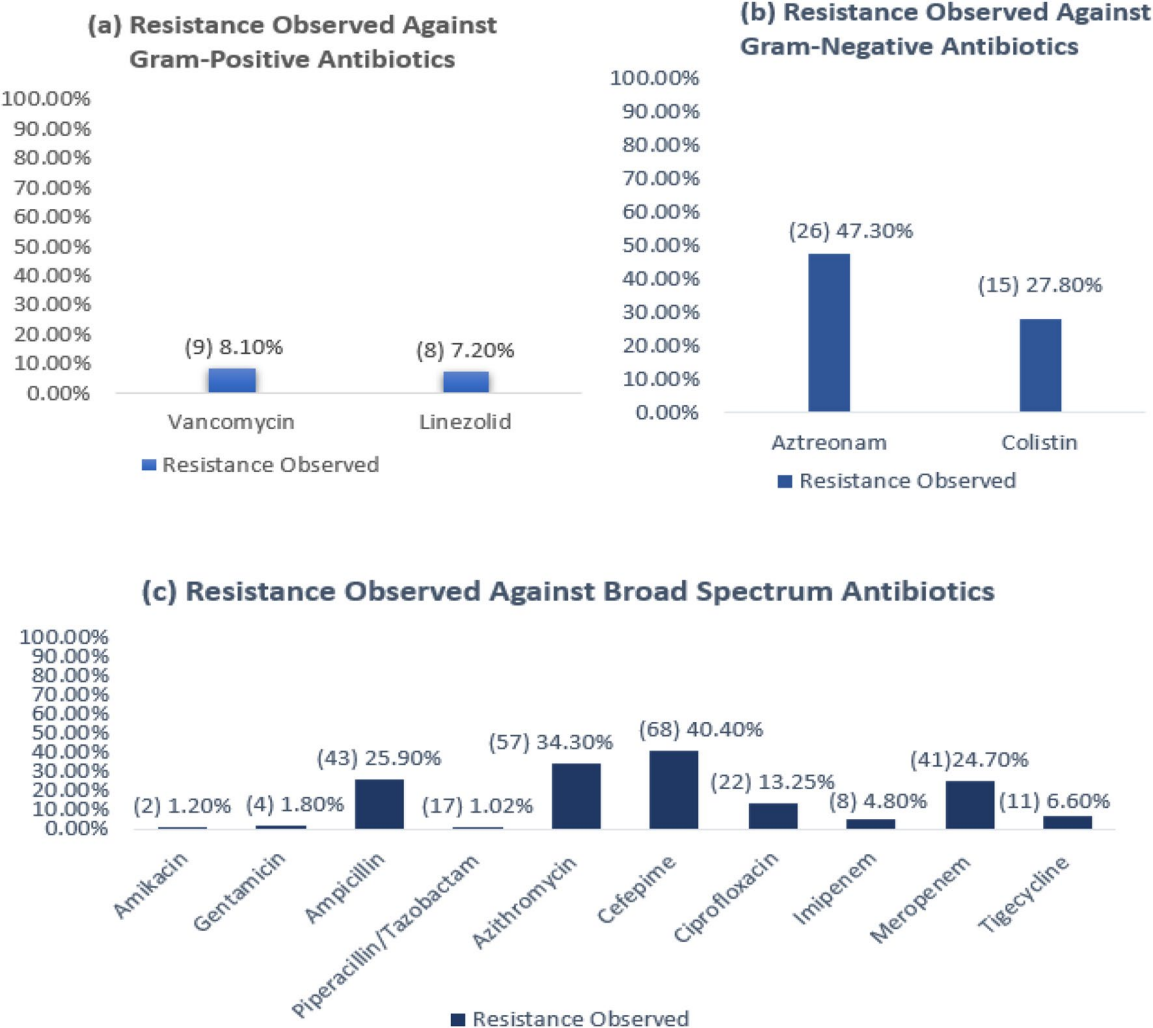
Percentage of resistance observed in all the isolates. On the left side of the figure, the number of isolates that showed resistance is mentioned in parenthesis. (**a**) Resistance observed against narrow-spectrum gram-positive antibiotics consisting of 111 isolates. (**b**) Resistance observed against narrow-spectrum gram-negative antibiotics consisting of 54 isolates. (**c**) Resistance observed against broad-spectrum antibiotics consisting of 165 isolates.

The broad-spectrum antibiotics that displayed the highest levels of resistance were ampicillin, azithromycin, cefepime, ciprofloxacin, and meropenem. For narrow-spectrum antibiotics, the highest levels of resistance were seen in aztreonam and colistin, which are narrow-spectrum antibiotics for Gram-negative bacteria (Fig. 1).

**Figure 2:**
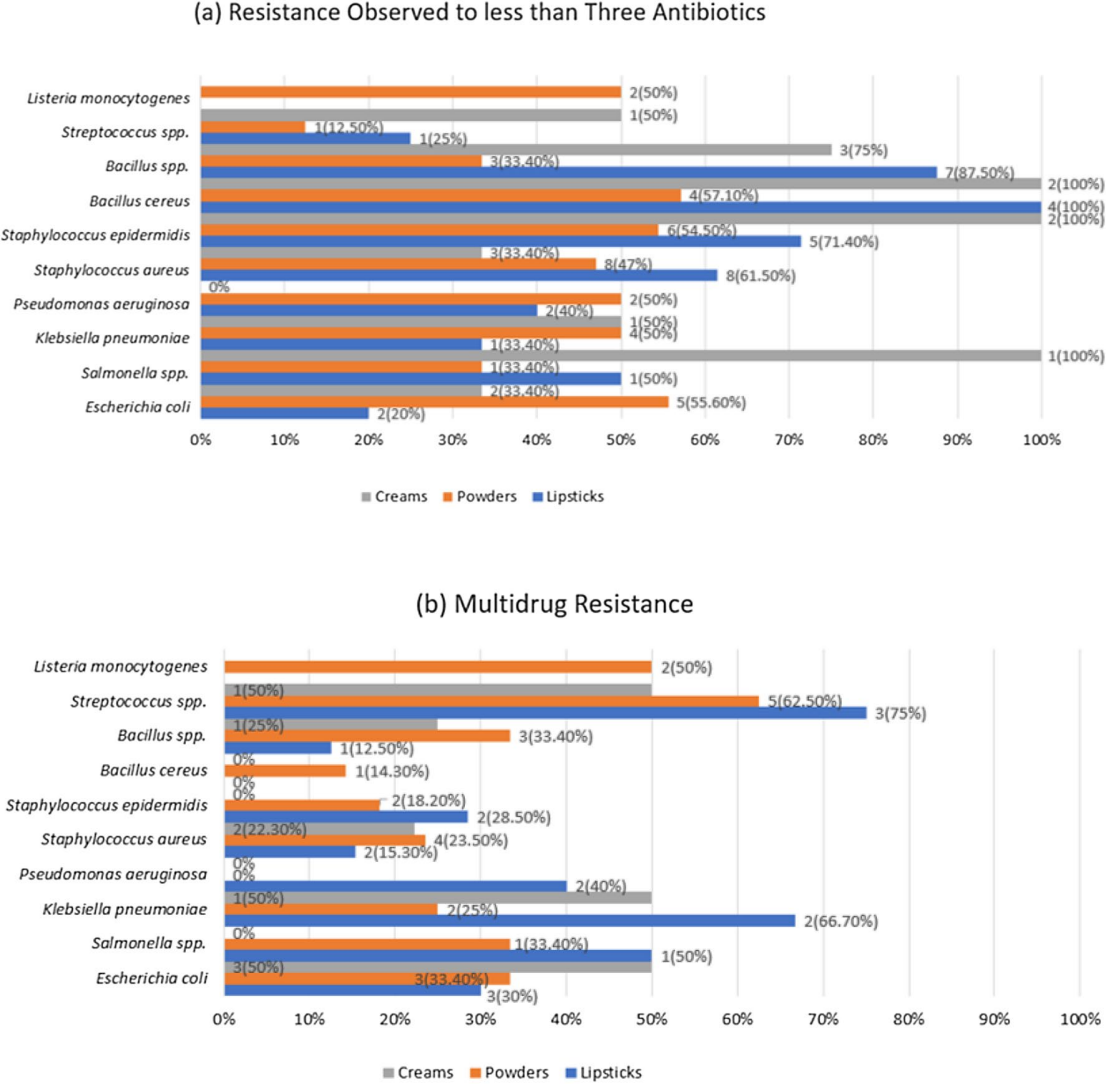
Antibiotic Susceptibility Assay. (**a**) Resistance observed in less than Three Antibiotics. (**b**)Multidrug Resistance. On the right side of the figure, the number of isolates that showed resistance is mentioned.

The majority of the Gram-negative and Gram-positive isolates showed resistance to less than three antibiotics and minority of the isolates exhibited multidrug resistance. *Pseudomonas aeruginosa* isolates obtained from cream were not resistant to any of the antibiotics. *Listeria monocytogenes* were only detected in powder samples. All other bacterial isolates were present in lipstick powder and cream samples. Also these bacterial isolates were shown to be resistant to less than three types of antibiotics (Fig. 2).

## Discussion

Despite the considerable amount of research conducted on the quality of pharmaceutical products in Bangladesh, there remains a dearth of information regarding the prevalence and impact of microbial contamination in locally manufactured products until recently^2,13,32^. According to the Bangladesh Standards and Testing Institution (BSTI),the International Organization for Standardization (ISO) and Food and Drug Administration (FDA), the level of contamination in cosmetic products should not exceed 10^3^ cfu/g or ml for non-eye cosmetics^3,4,6^. Our study has shown an alarming level of contamination in the cosmetic products tested (Table 1) which exceeds the acceptable limits of the BSTI, the ISO and FDA have provided^3,4^. Similar results were shown in recent studies denoting high levels of microbial contamination in the cosmetic products^2,25,32^.

For most of the gram-negative isolates, it was seen that the CFU/ml level was more than 10^3^. All Gram-negative isolates were detected in lipsticks, powders, and creams (Table 2). The Gram-negative isolates detected in the current study had also been reported in previous studies. In those studies, lipstick samples were seen to be contaminated with different bacteria such as *Staphylococcus aureus, Staphylococcus spp., Pseudomonas spp.* and *Citrobacter freundii* but not *Escherichia coli*^2,7–9^*.* Only in one study, *Escherichia coli* was isolated from lipstick samples, but it was found in only 0.6% of the collected lipstick samples^33^. This was also seen in the case of powder samples^2,10,11^. *Escherichia coli* was detected in creams in a previous study^14^. *Salmonella spp.* was previously detected^11^ in various eye cosmetics, powder, foundation and nail henna. *Klebsiella pneumoniae* was previously detected in creams and lotions^14^ and lip-gloss^16^. In previous studies, *Pseudomonas spp.* was isolated in creams^2^ and *Pseudomonas aeruginosa* was isolated in lipsticks^33^.

Similarly, in gram-positive isolates, most of the isolates showed CFU/ml of more than 10^3^ (Table 2), and the isolates detected in this study had been detected before in other studies. *Staphylococcus aureus* was previously detected in lipsticks^9^, powders^14^, lotions and creams^11^. *Staphylococcus epidermidis* was isolated in powders, blushers^11^, and various eye cosmetics^11,34,35^. *Bacillus cereus* isolates were previously detected in various eye cosmetics^24^, lip glosses^16^ and creams^15^. *Bacillus spp.* was previously detected in creams, lotions^12,14^ and eyeshadow^36^. In the current study, *Streptococcus spp.* isolates were detected in lipsticks and creams but not in powder samples(Table 2) and previously isolated in lip-gloss^16^, creams and lotions ^14^. *Listeria monocytogenes* was isolated only in powder samples (Table 2) and was detected in the past in various eye cosmetics^24^.

In this study, the level of bacterial load and type differs from sample to sample (Table 2). However, as previously mentioned, according to ISO 17516:2014 regulations, *Escherichia coli*, *Pseudomonas aeruginosa*, *Staphylococcus aureus* and *Candida albicans* must be completely absent in 1 ml or 1gm of the product^4,5^. So, even if a low bacterial load is detected of the aforementioned bacteria, the product will still be considered unsafe. Furthermore, according to the Bangladesh Standards and Testing Institution (BSTI) no pathogenic bacteria should be detected in cosmetics at any level^6^. Furthermore, Bangladesh can now export cosmetics to different Asian countries^37^. With that in mind it also needs to maintain the ASEAN (Association of Southeast Asian Nations) Cosmetic Directive. As per the ASEAN (Association of Southeast Asian Nations) Cosmetic Directive, the limit of total aerobic mesophilic microorganisms is less than 500 CFU/g or CFU/ml for products for children under 3 years, eye area and mucous membranes. For other products the limit of aerobic mesophilic organisms is less than 1000 CFU/g or CFU/ml. *Pseudomonas aeruginosa, Staphylococcus aureus* and *Candida albicans* must be absent in 0.1 g or 0.1 ml of the test sample^38^.

Preservatives may have been added to the cosmetics to reduce the microbial contamination but from our study they were largely ineffective as a high level of contamination was detected in most of the cosmetic products. The stability of preservatives depends on various factors such as solubility and partition in oil/water (O/W) or water/oil (W/O) emulsions, formulation pH, and temperature during use, and the volatility of the preservative. The product can become unpreserved in O/W emulsions and in lipophilic preservatives, such as parabens^39^. Again, preservatives control only the vegetative form of bacillus species, but do not kill their spores. To prevent bacillus contamination in a cosmetic product they should not be present in the raw materials meaning no soil or dust should get into the product^40^.

Hemolysis was observed in 66.7% of Gram-positive bacteria and 25% of Gram-negative bacteria. All the isolates of *Staphylococcus aureus, Bacillus cereus* and *Listeria monocytogenes* were seen to be beta hemolytic (Supplementary Fig. 6). In lipstick samples mostly beta hemolysis (61.9%) was observed. Alpha hemolysis (4.76%) was also observed to a lesser extent. None of the other types of cosmetics showed alpha hemolysis. Beta-hemolysis was observed in majority of all the samples. The lipstick samples showed the highest levels of beta hemolysis (61.9%), followed by cream (59.1%) and powder (56.36%) samples (Table 3). Since most of the cosmetic samples showed beta hemolysis, it is a cause for concern as hemolytic bacteria are most pathogenic for humans. These bacteria contain an endotoxin that can destroy red blood cells and hemoglobin.

*Staphylococcus aureus* isolates were further tested to assess their pathogenic capabilities. In DNase test, only 5.1% of the isolates were positive. Both isolates were obtained from powder samples. However, for coagulase test, 97.4% of the isolates tested positive for plasma coagulase-reacting factor (CRP). This correlates to a previous study^33^ where all the *Staphylococcus aureus* isolates were coagulase positive.

The antibiotics that displayed the highest levels of resistance were ampicillin, azithromycin, cefepime, ciprofloxacin, meropenem, aztreonam and colistin. The antibiotics that showed the lowest levels of resistance were amikacin, gentamicin, piperacillin/tazobactam, imipenem, tigecycline, linezolid and vancomycin (Fig. 2).

These findings partially corresponded with a previous study^41^ where both Gram-positive and Gram-negative isolates showed high resistance to ampicillin (34.5%), gentamicin (15.5%) and ciprofloxacin (14.3%). The levels of resistance showed by ampicillin and ciprofloxacin corresponded with this study but did not correspond to gentamicin. The resistance showed in tigecycline also corresponded with the present study. In the previous study, only Gram-negative isolates were tested against cefepime (9.7%), imipenem (9.7%), meropenem (6.45%), amikacin (6.45%), and colistin (3.2%). When compared with current findings, the resistance of cefepime, meropenem and colistin was seen to be less and the resistance was seen to be more in imipenem and amikacin. No resistance was observed against vancomycin, and linezolid (3.8%) showed a low level of resistance in the prior study, which did not correspond with our findings.

Most of the gram-negative and gram-positive bacteria detected were resistant to less than 3 antibiotics. In the case of *Escherichia coli,* multidrug resistance was within 30–50% (Fig. 2). The isolates showed varying levels of resistance to all the antibiotics used (Supplementary Fig. 7). In a previous study^41^ it was observed that antibiotics showing the highest resistance were to ampicillin and gentamicin. The isolates also showed resistance to other antibiotics such as cefepime, imipenem, piperacillin/tazobactam, amikacin, ciprofloxacin, and tigecycline.

For *Salmonella spp.* no multidrug resistance was observed in the isolates found in creams. Half of the isolates found in lipsticks and less than half of the isolates found in powder samples were found to be multidrug-resistant (Fig. 2). No isolates were seen to be resistant to ciprofloxacin, cefepime, piperacillin, gentamicin, and amikacin. The rest of the antibiotics tested showed low levels of resistance (Supplementary Fig. 8). Previous studies had isolated *Salmonella spp.*^2,42^ in cosmetics, but they were not tested for antibiotic susceptibility.

In the case of *Klebsiella pneumoniae,* multidrug resistance observed in the samples was within 12–67% (Fig. 2). All the antibiotics showed some level of resistance except for amikacin, gentamicin, and tigecycline (Supplementary Fig. 9). For *Pseudomonas aeruginosa,* multidrug resistance was observed only in the lipstick samples, which was 40% (Fig. 2). The isolates showed resistance to ampicillin, colistin, cefepime, azithromycin, aztreonam, and tigecycline (Supplementary Fig. 10). In a study conducted in 2021, *Klebsiella pneumoniae* isolates showed^41^ no resistance to gentamicin, amikacin, and tigecycline which was in accordance with the current study. In the same study, it was also seen that penicillins, carbapenems, and cephalosporins exhibited resistance; also, *Pseudomonas aeruginosa* isolates exhibited varying degrees of resistance to meropenem, imipenem, cefepime and ciprofloxacin.

For *Staphylococcus aureus,* multidrug resistance observed in the samples was between 15 and 24% (Fig. 2). Resistance was seen in cefepime, azithromycin, ciprofloxacin, vancomycin, linezolid, penicillins, and carbapenems (Supplementary Fig. 11). In *Staphylococcus epidermidis,* the isolates did not show multidrug resistance in cream samples, and for the other samples, a small portion of the isolates showed multidrug resistance (Fig. 2). Ampicillin, piperacillin/tazobactam, meropenem, cefepime, azithromycin, ciprofloxacin, and vancomycin were seen to be resistant (Supplementary Fig. 12). A prior study^41^ showed that *Staphylococcus aureus* isolates were resistant to gentamicin, ampicillin, ciprofloxacin, erythromycin and tigecycline. *Staphylococcus epidermidis* isolates were seen to be resistant to gentamicin, ampicillin, ciprofloxacin, erythromycin, vancomycin, linezolid, and tigecycline in the same study.

In the case of *Bacillus cereus*, multidrug resistance was observed in powder samples only, which was 14.3% (Fig. 2). Resistance was observed in amikacin, ampicillin, meropenem, cefepime, azithromycin, and vancomycin (Supplementary Fig. 13). A previous study^43^ confirmed these findings where the *Bacillus cereus* isolates showed resistance to ampicillin, cephalosporins and penicillin.

Multidrug resistance was 12–25% for *Bacillus spp.* (Fig. 2). The isolates showed resistance to ampicillin, piperacillin/tazobactam, meropenem, cefepime, azithromycin, ciprofloxacin, vancomycin, linezolid, and tigecycline (Supplementary Fig. 14). These findings did not correspond to a previous study^24^ where all the *Bacillus spp.* isolates were sensitive to every antibiotic tested.

*Streptococcus spp.* showed multidrug resistance for the majority of the isolates (Fig. 2). *Streptococcus spp.* isolates were resistant to penicillins, carbapenems, cefepime, azithromycin, vancomycin, linezolid, and tigecycline antibiotics (Supplementary Fig. 15). In a past study, *Streptococcus spp.*^9^ had been isolated, but the isolates were not tested for antibiotic susceptibility.

Multidrug resistance was observed in half of the samples of *Listeria monocytogenes* isolates. These isolates were only detected in powder samples (Fig. 2). Resistance was seen in ampicillin, cefepime, ciprofloxacin and vancomycin (Supplementary Fig. 16). This is in accordance with a study^24^ where *Listeria monocytogenes* was resistant to vancomycin and nalidixic acid.

In the present study, it can be observed that bacteria isolated from various cosmetics differed and a high level of contamination was present. According to ISO 2962:2010 lipsticks and powders are microbiologically low-risk products. This is because these products have a water activity below 75%, pH lower than 3 or higher than 10, or a quantity of alcohols higher than 20%^40^. However, from our study it could be seen that a high level of poly-contamination was seen in these products. This could be due to a myriad of reasons. The contamination could be present in the raw material or be present in the water used for formulating these products. Water is one of the most important factors in contamination of a product. The presence of *Escherichia coli* may be a sign of recent contamination by wastewater^18,44^. The product could have also been contaminated during manufacturing and packaging. Contamination can occur during the manufacturing process due to contact with operators, manufacturing equipment, and air. The cosmetic product is likely to be contaminated by human sources such as the part of the nasopharynx, the oral flora, the hair, the skin of the hands and even the intestinal flora. Bacteria such as fecal *Streptococci, Staphylococci, Enterobacteria*, and *Pseudomonas* can survive and even multiply in the product^45^. The equipment used to manufacture the products can also be a valid source of contamination. This could be due to maintenance materials (oils, grease), poor cleaning and product change. Air impurity could be another reason of contamination. Most of the air contamination (80%) occurs due to the number of workers together with the size of their movements^46^. A definitive reason for the cause of such high contamination in these products cannot be given unless the factories themselves are inspected.

Most of the isolates were not hemolytic. Majority of *Staphylococcus aureus* isolates possessed coagulase capabilities and a minority of *Staphylococcus aureus* isolates possessed DNase capabilities. The isolates were resistant to β-lactams, aminoglycosides, macrolides, fluoroquinolones, glycylcyclines, glycopeptides, oxazolidinones and polymyxin E. Diseases caused by such antibiotic resistant isolates could be challenging to treat and therefore a public health concern.

According to the FDA, the manufacturer is legally bound to ensure the quality of their cosmetics including keeping their products free of microbial contamination. The cosmetics should be checked for microbial contamination in every step of the production and distribution to avoid reaching high microbial content^32^. Manufacturers can avoid contamination of their products by assessing the quality of the raw material and water used while producing the cosmetics, keeping the equipment clean while maintaining a hygienic environment with proper handling of the cosmetics during production, storage, and distribution^1,4,13^. However, the findings of this study denote a lack of Good Manufacturing Practice (GMP) in the cosmetic manufacturing business in the Dhaka metropolitan area. In response to such practices numerous drives have taken place under the authority Bangladesh Standards and Testing Institution [BSTI]^28,29,47,48^. These measures have resulted in the factories being shut down, products being seized and being fined. In some cases the owners and employees of these factories have been imprisoned^48^. However, these measures are ineffective and from our study it can be observed that these products are still widely available. In 2023 a new bill named ‘Drugs and Cosmetics Bill, 2023’, was approved incorporating cosmetics into the jurisdiction of the proposed law, which was initially framed for regulating drugs. Considering the claims that fake and adulterated cosmetics have flooded the country’s market and effected the public health, the government decided to bring the production, import, marketing, and sale of cosmetics under the drugs law. Companies involved in any aspect of cosmetics will now require fresh licenses from the Directorate General of Drug Administration (DGDA). The punishment of manufacturing cosmetics without a license and producing fake cosmetics would be increased according to the new bill^49^. As the new bill has just been introduced it is not possible to say whether it would be effective in reducing the level of contamination of cosmetics. A way to reduce the level of contamination in cosmetic products could be to implement Good Manufacturing Practices. On the basis of our study, the level of contamination in local cosmetics is a potential public hazard. The healthcare policy makers and regulatory authorities should collaborate with the microbiology researchers and provide immediate attention to the local cosmetic industry, enforcing the guidelines to improve the quality of the cosmetic products and to avoid emergence of contaminated cosmetics induced diseases in Bangladesh.

## Materials and methods

### Sample collection

A total of 27 mass-marketed locally manufactured cosmetic samples were collected from different stores in the New Market and Tejgaon area of Dhaka, Bangladesh. All the samples were within the use-by date. Of the 27 samples lipsticks (n = 8), powders (n = 9), creams (n = 10) were collected. After collecting, they were transferred to the lab and subjected to microbiological analyses.

The cosmetics that were selected were leave-on products. These were microbiologically low-risk products. Rinse-off products like gels and shampoos have a high-water quantity of more than 75%. They also have a neutral pH which makes them suitable for microorganism development. They are microbiologically high-risk products according to ISO 29621:2010^40^. Leave-on cosmetic products are sometimes worn for the entire day and so they have longer contact with the skin and therefore are more likely to cause health problems. In comparison rinse-off products are kept usually rapidly washed away so even if microbial contamination was present, it would likely cause less harm than the leave-on products.

### Sampling handling and preliminary preparation

All of the microbiological analyses were performed according to the US Food and Drug Administration (FDA) Bacteriological Analytical Manual: Microbiological Methods for Cosmetics^3^. These methods were also followed in the case of handling the samples, as well as the preliminary preparation. The sample containers were inspected properly for any irregularities, and the surface was disinfected with 70% ethanol beforehand removing the contents. Then the surface was dried with tissues, and 1 g (ml) of the sample was weighed aseptically. Since powders, lipsticks, and creams have different compositions, different processes were used for their initial preparation.

#### For powder samples

For powders, 1 g of sample was aseptically removed from the container and inserted in a test tube containing 1 ml sterile Tween 80, followed by the addition of 8 ml sterile Modified Letheen Broth (MLB) (HiMedia Laboratories). The mixture was vortexed for homogenization and counted as the 10^–1^ dilution.

#### For cream and lipstick samples

For creams and lipsticks, 1 g of sample was aseptically removed from the container and inserted in a test tube containing 1 ml sterile Tween 80 and five to seven glass beads. The total contents were homogenized with the help of a vortex mixture. Then, 8 ml of sterile MLB was added to adjust the total volume to 10 ml and mixed adequately for the 10^–1^ dilution.

### Aerobic plate count (APC)

Aerobic plate count was done using the spread plate method on Modified Letheen Agar (MLA). The preparation was diluted decimally in MLB to get discreet countable colonies for the count. From the inoculum, 0.1 ml was spread on MLA with a sterile spreader in an aseptic way and incubated for 48 h at 30 ± 2 °C.

For calculating aerobic plate count, the FDA’s Bacteriological Analytical Manual: Aerobic Plate Count was followed^50^*.*

•For plates with 25–250 CFU:

1 $$N = \frac{\sum C}{{\left[ {\left( {1 \times n1} \right) + \left( {0.1 \times n2} \right)} \right] \times \left( d \right)}}$$

where N = Number of colonies per ml or gram of the cosmetic product, Σ C = Sum of all the colonies from all plates counted, n1 = Number of plates in the first dilution counted, n2 = Number of plates in the second dilution counted, d = Dilution from which the first counts were obtained.

- For plates with fewer than 25 CFU

If plates from both dilutions contain less than 25 CFU each, the actual plate count should be recorded, but the count should be counted as less than

2 $$N = 25{ } \times { }1/{\text{d}}$$

where N = Number of colonies per ml or gram of the cosmetic product, d = the dilution factor for the dilution from which the first counts were obtained.

- For plates with more than 250 CFU

If plates from both dilutions are more than 250 CFU each (but fewer than 100/cm^2^), estimate the aerobic counts from the plates (EAPC) nearest 250 and multiply by the dilution. So the equation is

3 $$N = 250 \times d$$

where N = Number of colonies per ml or gram of the cosmetic product, d = the dilution factor for the dilution from which the first counts were obtained.

### Bacteria culture and identification

To identify the presence of target microorganisms, 0.1 ml of each dilution was spread on different selective media and incubated for 48 h at 30 ± 2 °C. After incubation, the primary identification was made based on colony morphology and gram staining. The different selective media that were used and the colony morphology are presented in Supplementary Table 1. This procedure was done following the FDA’s Bacteriological Analytical Manual: Microbiological Methods for Cosmetics^3^.

### Biochemical tests

Further identification was made by biochemical tests, which include motility-indole-urease test (MIU), catalase test, methyl red test, Vogues Proskauer test, oxidase test, triple sugar iron test, and citrate utilization test. The criteria for the interpretation of biochemical tests are presented in Supplementary Table 2. *Escherichia coli, Klebsiella pneumoniae, Staphylococcus aureus, Streptococcus spp*. and *Salmonella spp.* Bacteria were further identified by Polymerase Chain Reaction (PCR).

### Hemolysis test

Blood agar plate was used to observe if the bacterial isolates could lyse the red blood cells and digest the hemoglobin. This test was also used for bacterial identification. The isolates possessing hemolysins created a clear zone (Alpha hemolysis) or partially clear zone (Beta hemolysis) in the blood agar. No clear zone (Gamma hemolysis) indicates no lysis of red blood cells.

### Bacterial DNA extraction and amplification

The bacteria were inoculated into Nutrient Agar and were incubated at 37 °C for 24 h. A single colony of the bacteria was selected. Then, a single colony of bacteria was added to 200 µl of nuclease-free water. This was done for every bacterium that was identified using the PCR method. These samples were then boiled at 95 °C for 20 min and then cooled at − 20 °C for 5 min. After that, the samples were centrifuged for 10 min at 5000 × g. The DNA of *Escherichia coli, Klebsiella pneumoniae, Staphylococcus aureus, Streptococcus spp*. and *Salmonella spp.* were obtained in this way. The bacterial DNA samples were stored at − 20 °C.

For each bacterial sample, 4 µl template DNA, 12.5 µl Master Mix, 4.5 µl of nuclease-free water, 2 µl forward primer and 2 µl reverse primer were adjusted to be a 25 µL of final solution for PCR. The primers, PCR thermocycler conditions and amplicon size is mentioned in Supplementary Table 3. The PCR products were examined by electrophoresis in a 2% agarose gel using 1X TAE buffer, stained with Midori Green Advance stain as well as ethidium bromide. The products were observed under a UV transilluminator.

### Antibiotic susceptibility test

This test was done following the Kirby-Bauer disc diffusion protocol, and the zones of inhibition were interpreted according to the CLSI standards published in 2018. The list of antibiotics used, their group, effectiveness, disc potency and interpretive criteria are presented in Supplementary Table 4. Multidrug resistance (MDR) was defined as non-susceptibility to at least one agent in three or more antimicrobial categories following the definition by Clinical Laboratory Standards Institute (CLSI), the European Committee on Antimicrobial Susceptibility Testing (EUCAST), and the United States Food and Drug Administration (FDA)^51^.

### Coagulase test

For tube coagulase tests, colonies of *Staphylococcus aureus* isolates were re-suspended in 300 µl of diluted rabbit plasma. Two-fold dilution was performed on the rabbit plasma with physiological saline. The tubes were incubated at 35 °C for 1 h and observed for clot formation.

### DNase test

DNase test was performed by incubating the *Staphylococcus aureus isolates* for 24 h at 37 °C on DNase agar containing toluidine blue dye. Clear zones around the bacterial colonies indicated DNase positive colonies.

### Flowchart of the methods used in this study

**Figure 3:**
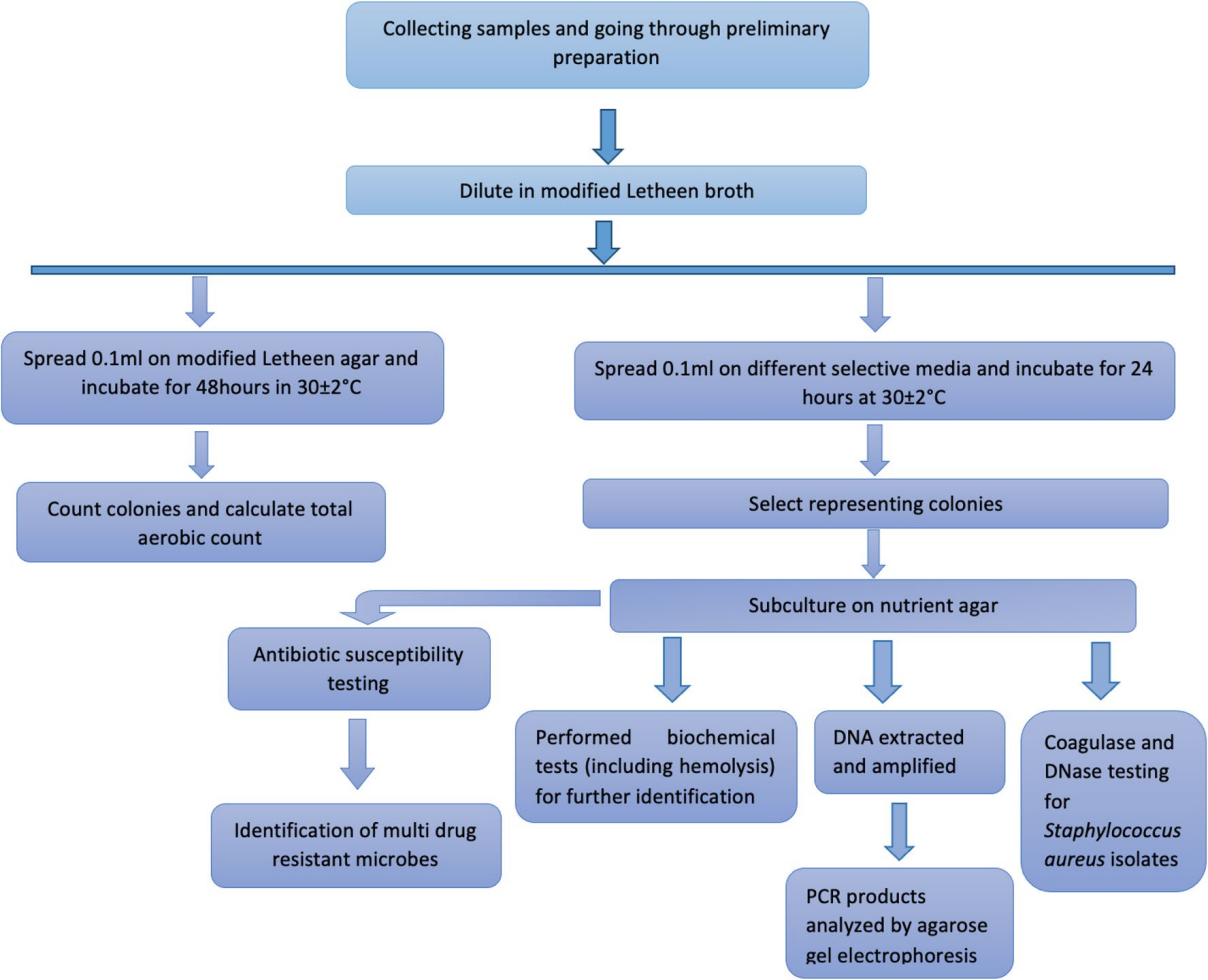
Flowchart detailing the methods used in this study.

The methods that were followed in this study are shown as a flowchart in Fig. 3.

## Supplementary Information

Supplementary Information.

## Data Availability

All data generated or analyzed during this study are included in this published article (and its Supplementary Information files).
