# Supplementary material for: Assessment of potential pathogenic bacterial load and multidrug resistance in locally manufactured cosmetics commonly used in Dhaka metropolis
Source: Sci Rep. 2023 May 13;13:7787. doi: 10.1038/s41598-023-34782-9 (PMC10182989; doi:10.1038/s41598-023-34782-9)
Supplement: Supplementary file 1 — Supplementary Information. [file 41598_2023_34782_MOESM1_ESM.docx]

**Supplementary Materials**

**Supplementary Table** **1: Colony Morphology of Specific Bacteria on Selective Media**

| Organism | Gram Positive/Negative | Media | Expected colony morphology |
| --- | --- | --- | --- |
| *Bacillus cereus* | Gram-positive | HiCrome Bacillus Agar | light blue, large, flat colonies with a blue center |
| *Listeria monocytogenes* | Gram-positive | Listeria Selective Oxford Agar Base Media | positive reaction for esculin hydrolysis, blackening of the medium around the colony |
| *Staphylococcus aureus* | Gram-positive | MSA Agar | yellow/white colonies surrounded by yellow zone |
| *Staphylococcus epidermidis* | Gram-positive | MSA Agar | red |
| *Streptococcus spp.* | Gram -positive | KF Streptococcus Agar Base | red-maroon colonies with yellow zone |
| *Escherichia coli* | Gram-negative | EMB Agar | purple with a black center and green metallic sheen |
| *Salmonella spp.* | Gram-negative | XLD Agar | pink-red with black centers |
| *Pseudomonas aeruginosa* | Gram-negative | Cetrimide Agar | yellow-green glow under UV ray |
| *Klebsiella pneumoniae* | Gram-negative | HiCrome KPC Agar (without added antibiotic) | bluish green |

**Supplementary Table** **2: Biochemical Test Interpretation for Different Organisms**

| Organism | Motility | Indole | Urease | Catalase | Oxidase | MR | VP | Citrate utilization | Glucose  Ferm. | Sucrose  Ferm. | Lactose  Ferm. | Gas Prod. | H_2_S  Prod. | Hemolysis |
| --- | --- | --- | --- | --- | --- | --- | --- | --- | --- | --- | --- | --- | --- | --- |
| *Bacillus cereus* | +ve | -ve | +ve | +ve | -ve | -ve | +ve | +ve | +ve | -ve | -ve | . | +ve | Beta |
| *Bacillus spp.* | +ve | -ve | +ve | -ve | +ve | -ve | -ve | +ve | +ve | -ve | +ve | +ve | -ve | Alpha/ Beta/Gamma |
| *Listeria monocytogenes* | +ve | -ve | -ve | +ve | -ve | +ve | +ve | -ve | +ve | +ve | +ve | -ve | -ve | Beta |
| *Staphylococcus aureus* | -ve | -ve | +ve | +ve | -ve | +ve | +ve | +ve | +ve | +ve | +ve | -ve | -ve | Beta |
| *Staphylococcus epidermidis* | -ve | +ve | +ve | +ve | -ve | -ve | +ve | -ve | +ve | +ve | +ve | +ve | +ve | Gamma |
| *Streptococcus spp.* | -ve | -ve | -ve | -ve | -ve | +ve | -ve | +ve | +ve | +ve | +ve | . | -ve | Beta |
| *Escherichia coli* | +ve | +ve | -ve | +ve | -ve | +ve | -ve | -ve | +ve | variable | +ve | +ve | -ve | Alpha/  Beta/  Gamma |
| *Salmonella spp.* | +ve | -ve | -ve | +ve | -ve | +ve | -ve | -ve | +ve | -ve | -ve | +ve | +ve | Gamma |
| *Pseudomonas aeruginosa* | +ve | -ve | -ve | +ve | +ve | -ve | -ve | +ve | -ve | -ve | -ve | +ve | -ve | Beta |
| *Klebsiella pneumoniae* | -ve | -ve | +ve | +ve | -ve | -ve | +ve | +ve | +ve | +ve | +ve | +ve | -ve | Gamma |

**Supplementary Table 3: Primers and PCR Conditions used in this Study**

| Name of bacteria | Primer Designation | Primer Sequence | Product size | PCR conditions |
| --- | --- | --- | --- | --- |
| *Salmonella* *spp* | *inv*A -F | 5′-GTGAAATTATCGCCACGTTCGGGCAA-3' | 284bp | An initial incubation at 72°C for 7 min followed by 35 cycles of denaturation at 94° C for 1 min; primer annealing at 53 ° C for 2 min; primer extension at 72°C for 3 min. Following the last cycle, there was a 7 min incubation at 72 ° C. |
|  | *inv*A -R | 5′-TCATCGCACCGTCAAAGGAACC-3′ |  |  |
| *Klebsiella pneumoniae* | KP Pf-F: | 5′-ATTTGAAGAGGTTGCAAACGAT-3′ | 130 bp | The cycling conditions were 10 min at 94 °C followed by 35 cycles of 30s at 94 °C, 20s at 57 °C and 20s at 72 °C, followed by a 10 min hold at 72 °C. |
|  | KP Pr1-R: | 5′-TTCACTCTGAAGTTTTCTTGTGTTC-3′ |  |  |
| *Escherichia coli* | ECO- F | 5′-GACCTCGGTTTAGTTCACAGA-3′ | 585 bp | Initial denaturation at 95°C for 5 min; 35 cycles of denaturation at 94°C for 45s, annealing at 45°C for 45s and extension for 1 min followed by a final extension at 72°C for 5 min. |
|  | ECO-R | 5′-CACACGCTGACGCTGACCA-3′ |  |  |
| *Staphylococcus aureus* | Nuc - F | 5'-GCG ATT GAT GGT GAT ACG GT-3' | 279bp | Initial denaturation was performed at 95°C for 5 min, followed by 30 cycles of denaturation at 95°C for 1 min, annealing temperature of primers was 55°C for 45s and extension at 72°C for 1 min. The final extension was conducted at 72°C for 10 min. |
|  | Nuc -R | 5'-AGC CAA GCC TTG ACG AAC TAA AGC-3' |  |  |
| *Streptococcus spp.* | Str1-F | 5’-GTACAGTTGCTTCAGGACGTATC-3' | 137 bp | Initial denaturation at 94°C for 10 min, 40 cycles of denaturation at 95°C for 15 sec, annealing temperature 60°C for 1min and extension at 65°C for 1sec. The final extension was at 72°C for 3 min. |
|  | Str2- R | 5’-ACGTTCGATTTCATCACGTTG-3' |  |  |

**Supplementary Table** **4: List of Antibiotics used in the Experiment**

| Serial no | Antibiotic | Group | Effective against | Disc code | Disc potency (µg) | Interpretative Criteria | | |
| --- | --- | --- | --- | --- | --- | --- | --- | --- |
|  |  |  |  |  |  | Sensitive mm or more | Intermediate mm | Resistant mm or less |
| 1 | Gentamicin | Aminoglycoside | Gram-positive and Gram-negative | GEN | 10 | 15 | 13-14 | 12 |
| 2 | Amikacin | Aminoglycoside | Gram-positive and Gram-negative | AK | 30 | 17 | 15–16 | 14 |
| 3 | Ampicillin | Beta-lactamase | Gram-positive and Gram-negative | AMP | 10 | 17 | 14–16 | 13 |
| 4 | Meropenem | Carbapenem | Gram-positive and Gram-negative | MEM | 10 | 23 | 20–22 | 19 |
| 5 | Imipenem | Carbapenem | Gram-positive and Gram-negative | IMI | 10 | 23 | 20–22 | 19 |
| 6 | Cefepime | Cephalosporin | Gram-positive and Gram-negative | CPM | 30 | 25 | 19–24 | 18 |
| 7 | Piperacillin tazobactam | Penicillin and beta-lactamase inhibitor | Gram-positive and Gram-negative | PIT | 100/10 | 21 | 18–20 | 17 |
|  |  |  |  |  |  |  |  |  |
| 8 | Azithromycin | Macrolide | Gram-positive and Gram-negative | AZM | 15 | 18 | 14–17 | 13 |
|  |  |  |  |  |  |  |  |  |
| 9 | Ciprofloxacin | Fluoroquinolone | Gram-positive and Gram-negative | CIP | 5 | 21 | 16–20 | 15 |
| 10 | Tigecycline | Glycylcyline | Gram-positive and Gram-negative | TGC | 15 | 18 | 15-17 | 15 |
| 11 | Vancomycin | Glycopeptide | Gram-positive | VA | 30 | 17 | 15–16 | 14 |
| 12 | Linezolid | Oxazolidinones | Gram-positive | LZ | 30 | 23 | 21–22 | 20 |
| 13 | Aztreonam | Monobactam | Gram-negative | AT | 30 | 21 | 18–20 | 17 |
| 14 | Colistin | Polymyxin E | Gram-negative | CT | 10 | - | 11-17 | - |


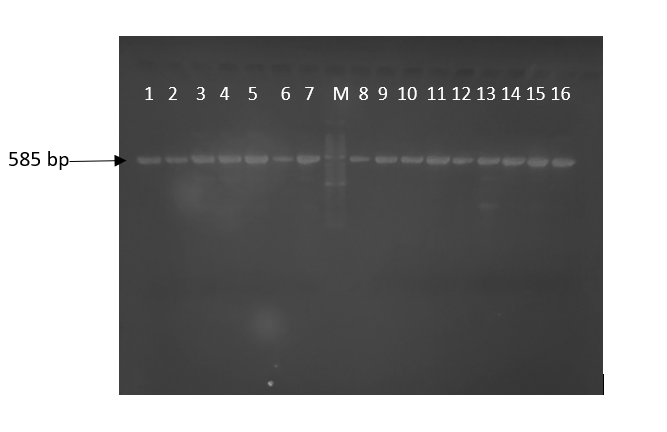


**Supplementary Figure 1:** **Agarose gel electrophoresis of PCR assay of Escherichia coli isolates.** Here Lane (M) is 50bp DNA marker, and Lane (1-16) are some positive samples at 585bp.

**
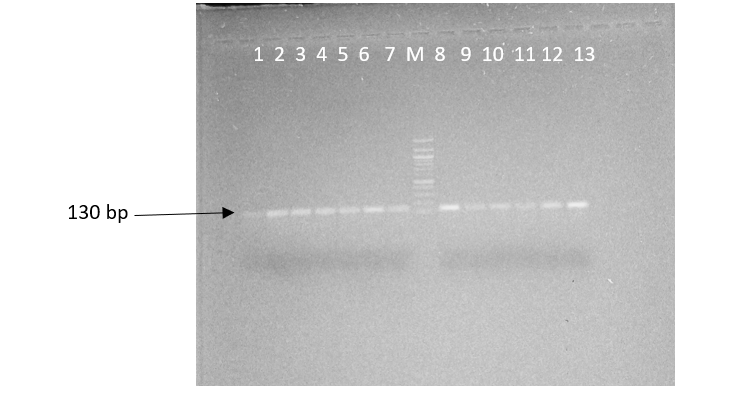
**

**Supplementary Figure 2:**  **Agarose gel electrophoresis of PCR assay of Klebsiella pneumoniae isolates.** Here Lane (M) is 100 bp DNA marker, and Lane (1-13) are some positive samples at 130bp.


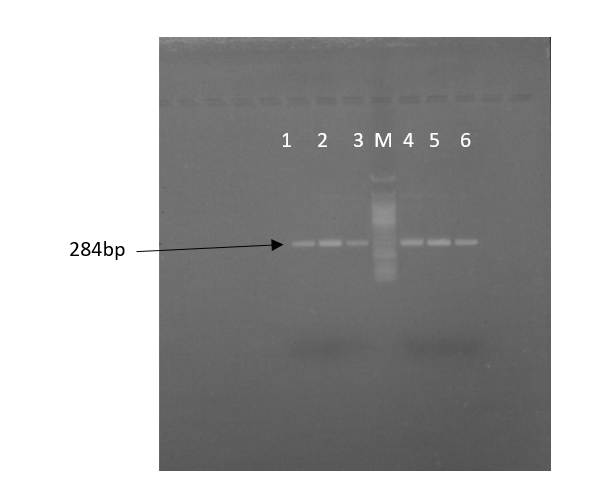


**Supplementary Figure 3**:  **Agarose gel electrophoresis of PCR assay of Salmonella spp. isolates.** Here Lane (M) is 50bp DNA marker, and Lane (1-6) are some positive samples at 284bp.

**
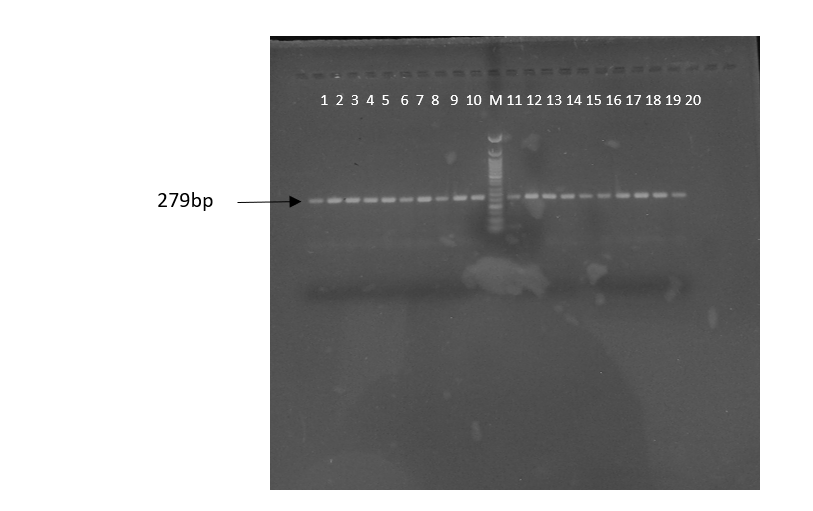
**

**Supplementary Figure 4:**  **Agarose gel electrophoresis of PCR assay of Staphylococcus aureus isolates.** Here Lane (M) is 50bp DNA marker, and Lane (1-20) are some positive samples at 279bp.

**
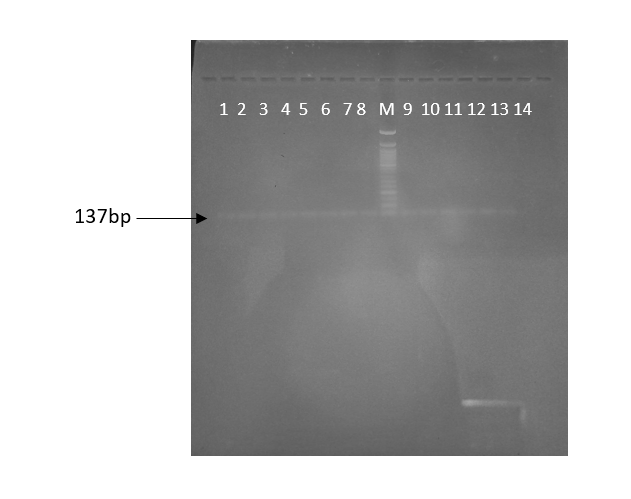

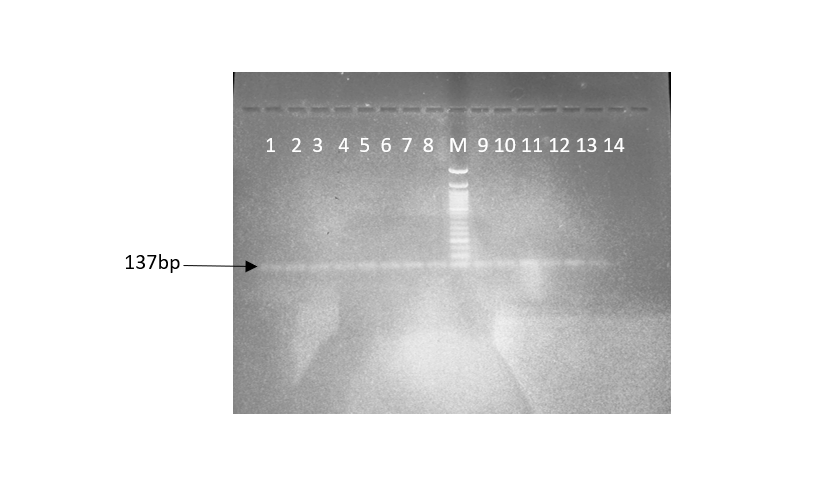
**

**Supplementary Figure 5:** **Agarose gel electrophoresis of PCR assay of Streptococcus spp. isolates.** On the left is the original photo which is grainy. On the right is a high-contrast image for easier viewing. Here Lane (M) is 100bp DNA marker and Lane (1-14) are some positive samples at 137bp.

**
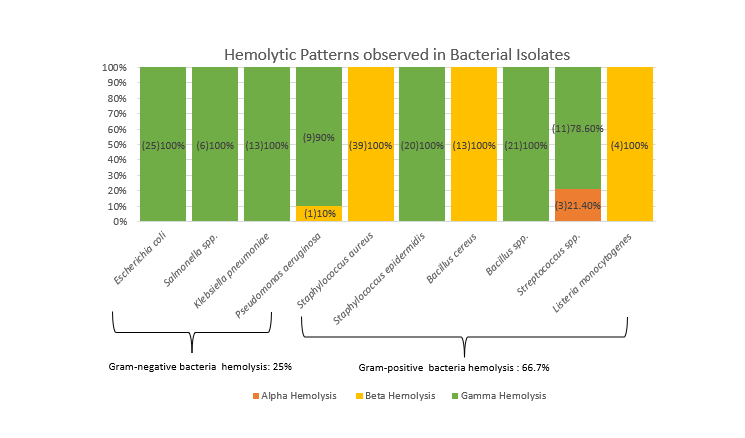
**

**Supplementary Figure 6: Hemolytic Patterns Observed in Bacterial Isolates.** On the left side of the figure, the number of isolates that showed resistance is mentioned in parenthesis.

**Supplementary Figure** **7: Resistance Observed in Escherichia coli.** On the right side of the figure, the number of isolates that showed resistance is mentioned in parenthesis.

**Supplementary** **Figure 8: Resistance Observed in Salmonella spp.** On the right side of the figure, the number of isolates that showed resistance is mentioned in parenthesis.

**Supplementary Figure** **9: Resistance Observed in Klebsiella pneumoniae.** On the right side of the figure, the number of isolates that showed resistance is mentioned in parenthesis.

**Supplementary Figure** **10: Resistance Observed in Pseudomonas aeruginosa.** On the right side of the figure, the number of isolates that showed resistance is mentioned in parenthesis.

**Supplementary Figure** **11: Resistance Observed in Staphylococcus aureus.** On the right side of the figure, the number of isolates that showed resistance is mentioned in parenthesis.

**Supplementary Figure** **12: Resistance Observed in Staphylococcus epidermidis.**  On the right side of the figure, the number of isolates in which resistance was found is mentioned in parenthesis.

**Supplementary Figure** **13: Resistance Observed in Bacillus cereus.** On the right side of the figure, the number of isolates that showed resistance is mentioned in parenthesis.

**Supplementary Figure** **14: Resistance Observed in Bacillus spp.** On the right side of the figure, the number of isolates that showed resistance is mentioned in parenthesis.

**Supplementary Figure** **15: Resistance Observed in Streptococcus spp.** On the right side of the figure, the number of isolates that showed resistance is mentioned in parenthesis.

**Supplementary Figure** **16: Resistance Observed in Listeria monocytogenes.** On the right side of the figure, the number of isolates that showed resistance is mentioned in parenthesis.

**Legends:**

- **Supplementary Table 1:** Colony Morphology of Specific Bacteria on Selective Media. This table indicates the type of media used to grow a specific type of bacteria and the expected morphological colonies to be observed on that media.
- **Supplementary Table 2:** Biochemical Test Interpretation for Different Organisms. This table mentions the biochemical nature of specific bacteria.
- **Supplementary Table 3:** Primers and PCR Conditions used in this Study. This table mentions the name of the bacteria, the name of the primer used, its sequence, expected product size and the PCR conditions needed for PCR to be successful.
- **Supplementary Table 4:** List of Antibiotics used in the Experiment. Mentions the type of antibiotic group, its spectrum, potency, and interpretive criteria.
- **Supplementary Figure 1:** Agarose gel electrophoresis of PCR assay of *Escherichia coli* isolates. Lane (M) is 50bp DNA marker, and Lane (1-16) are the positive samples at 585bp.
- **Supplementary Figure 2:** Agarose gel electrophoresis of PCR assay of *Klebsiella pneumoniae* isolates. Lane (M) is 100 bp DNA marker, and Lane (1-13) are the positive samples at 130bp.
- **Supplementary Figure 3:** Agarose gel electrophoresis of PCR assay of *Salmonella spp.* isolates. Lane (M) is 50bp DNA marker, and Lane (1-6) are positive samples at 284bp.
- **Supplementary Figure 4:** Agarose gel electrophoresis of PCR assay of *Staphylococcus aureus* isolates. Lane (M) is 50bp DNA marker, and Lane (1-20) are positive samples at 279bp.
- **Supplementary Figure 5:** Agarose gel electrophoresis of PCR assay of *Streptococcus spp*. isolates. On the left is the original photo which is grainy and the bands are difficult to decipher. On the right is a high-contrast image for easier viewing. Lane (M) is 100bp DNA marker and Lane (1-14) are positive samples at 137bp.
- **Supplementary Figure 6:** Hemolytic Patterns Observed in Bacterial Isolates. On the left side of the figure, the number of isolates that showed resistance is mentioned in parenthesis.
- **Supplementary Figure 7:** Resistance Observed in *Escherichia coli*. On the right side of the figure, the number of isolates that showed resistance is mentioned in parenthesis. The antibiotics tested on the isolates are also mentioned.
- **Supplementary Figure 8:** Resistance Observed in *Salmonella spp*. On the right side of the figure, the number of isolates that showed resistance is mentioned in parenthesis. The antibiotics tested on the isolates are also mentioned.
- **Supplementary Figure 9:** Resistance Observed in *Klebsiella pneumoniae*. On the right side of the figure, the number of isolates that showed resistance is mentioned in parenthesis. The antibiotics tested on the isolates are also mentioned.
- **Supplementary Figure 10:** Resistance Observed in *Pseudomonas aeruginosa*. On the right side of the figure, the number of isolates that showed resistance is mentioned in parenthesis. The antibiotics tested on the isolates are also mentioned.
- **Supplementary Figure 11:** Resistance Observed in *Staphylococcus aureus*. On the right side of the figure, the number of isolates that showed resistance is mentioned in parenthesis. The antibiotics tested on the isolates are also mentioned.
- **Supplementary Figure 12:** Resistance Observed in *Staphylococcus epidermidis*. On the right side of the figure, the number of isolates in which resistance was found is mentioned in parenthesis. The antibiotics tested on the isolates are also mentioned.
- **Supplementary Figure 13:** Resistance Observed in *Bacillus cereus*. On the right side of the figure, the number of isolates that showed resistance is mentioned in parenthesis. The antibiotics tested on the isolates are also mentioned.
- **Supplementary Figure 14:** Resistance Observed in *Bacillus spp*. On the right side of the figure, the number of isolates that showed resistance is mentioned in parenthesis. The antibiotics tested on the isolates are also mentioned.
- **Supplementary Figure 15:** Resistance Observed in *Streptococcus spp*. On the right side of the figure, the number of isolates that showed resistance is mentioned in parenthesis. The antibiotics tested on the isolates are also mentioned.
- **Supplementary Figure 16:** Resistance Observed in *Listeria monocytogenes*. On the right side of the figure, the number of isolates that showed resistance is mentioned in parenthesis. The antibiotics tested on the isolates are also mentioned.
